# A New Microstructural Concept and Water-Free Manufacturing of an Al_2_O_3_-Based Refractory Material for Auxiliary Equipment of Sintering Furnaces

**DOI:** 10.3390/ma18174144

**Published:** 2025-09-04

**Authors:** Monika Spyrka, Piotr Kula, Sebastian Miszczak

**Affiliations:** Institute of Materials Science and Engineering, Lodz University of Technology, Stefanowskiego 1/15, 90-924 Lodz, Poland; piotr.kula@p.lodz.pl (P.K.); sebastian.miszczak@p.lodz.pl (S.M.)

**Keywords:** spherical alumina, Al_2_O_3_, refractory material, ceramic composite, dry forming refractory, porosity, mechanical strength, uniaxial pressing, thermal resistance

## Abstract

This study presents the development of a novel alumina-based ceramic composite designed for refractory applications in auxiliary components of sintering furnaces. The innovative concept relies on a three-phase microstructural architecture: a fine-grained alumina matrix improves cohesion, coarse particles act as crack propagation barriers, and spherical granules are intentionally introduced to increase porosity while preserving mechanical strength. This design reduces thermal capacity, enhancing the material’s energy efficiency under high-frequency thermal cycling and offering potential for operating cost reduction. A further novelty is the water-free forming process, which eliminates issues related to drying and deformation. The material was characterized using scanning electron microscopy (SEM), mechanical strength testing, and refractoriness under load (RUL) analysis to establish the structure–property relationships of the developed composite. The results demonstrate that the developed spherical alumina-based composite possesses excellent thermal and mechanical properties, making it a promising candidate for high-temperature industrial applications, particularly as auxiliary refractory plates.

## 1. Introduction

In the sintering processes of oxide ceramics, there is a need to use tooling (e.g., auxiliary plates) made of durable refractory materials. Traditional refractory ceramic materials, despite their strength, show limitations in terms of resistance to extreme working conditions [1,2,3]. Deformation, structural cracks and loss of performance at high temperatures and cyclic thermal loads are common problems [4,5]. In addition, the dynamic development of the industry presents manufacturers with a constant need to improve production efficiency, reduce costs, energy consumption and environmental impact [6,7,8].

Currently, literature reports describe the state of the art of new refractory materials, which focuses on structural modifications to create composites with enhanced properties [9,10,11]. Research focuses on increasing mechanical strength through additives that improve resistance to cracking and wear, while achieving lightness, porosity, fire resistance at reasonable production and operating costs [9,12,13]. While alumina-based materials have been extensively studied for various functional applications, including core–shell phosphor systems [14], their use in structurally porous refractory composites remains limited. This study introduces a novel concept that combines closed porosity and mechanical integrity in a dry-formed, alumina-based structure designed for thermal cycling environments.

The concept of the developed composite material is based on the use of spherical Al_2_O_3_ as a pore-forming phase in a matrix of solid, fine-grained Al_2_O_3_ ceramics with inclusions of large alumina grains as blockers for crack propagation. The first attempts to make such a structured ceramic composite were carried out using the technique of slip casting, drying and then sintering [15]. During preliminary tests, several technological problems were identified, such as inhomogeneities in the distribution of the individual morphological forms of Al_2_O_3_ in the final microstructure, long drying times after slip casting as well as deformations and cracks occurring during drying and sintering [15].

The paper describes an alternative technology for the manufacture of lightweight ceramic composite based on spherical Al_2_O_3_, employing a uniaxial compression molding, which eliminates the use of water and drying process. The results of research into the selection of raw materials, the optimization of parameters in the pressing and sintering stages, and the structure and mechanical properties of the resulting composite are described.

## 2. Materials and Methods

### 2.1. Manufacturing Process of the Refractory Material

#### 2.1.1. Preliminary Assumptions

Refractory materials, due to the specific nature of their applications, must meet a range of often conflicting requirements. In developing the technology for a new refractory composite material, based on existing material and technological solutions, the following initial assumptions were adopted:

- capability to operate at high temperatures (around 1700 °C);
- reduction of density and heat capacity;
- retention of mechanical strength (high-temperature strength);
- improvement of the manufacturing process (reduction of shrinkage and cracking).

Al_2_O_3_ was selected as the primary raw material for the production of the new type of ceramic refractory material. Due to its properties, such as high abrasion resistance, stability at elevated temperatures, hot strength and corrosion resistance [16], it is a widely used refractory material [3].

A critical analysis of the available scientific literature revealed that the final properties of refractory materials largely depend on the selection of raw materials with appropriate granulometry and particle shape [17]. The particle size distribution of the applied powders—including both fine and coarse fractions—as well as their surface morphology, plays a crucial role in the design of ceramic composites intended for high-temperature applications [18].

Fine-grained powders, characterized by a high specific surface area, ensure good particle packing within the matrix, resulting in greater green body density and improved mechanical properties after sintering [19]. Additionally, the presence of fine fractions promotes the formation of diffusion necks between particles, which enhance material cohesion [20]. On the other hand, coarse-grained aggregates act as structural reinforcements and can effectively hinder crack propagation by mechanisms such as deflection, arrest, or branching of the crack front [21,22].

Of particular interest is the use of spherical forms of alumina particles as a composite component [23,24]. Compared to irregular particles, spheres exhibit superior rheological properties during forming processes, promoting uniform distribution within the matrix and minimizing defects caused by localized stresses. The spherical shape may also contribute to reducing the concentration of mechanical stresses in the material, thereby decreasing the risk of microcrack formation during high-temperature service and under cyclic thermal loads. Furthermore, the use of hollow spheres—with their closed structure—allows for the introduction of controlled closed porosity into the material structure without significantly compromising its mechanical properties. This approach enables the development of a composite material that is simultaneously lightweight, refractory, and resistant to thermal fatigue. The synergistic effect resulting from the combination of fine and coarse fractions, alongside the incorporation of spherical particles, forms the foundation for the development of a new type of ceramic material suitable for operation under the most demanding industrial conditions.

The results of previous studies, as well as gaps identified in the scientific literature, confirm the rationale for developing a ceramic composite material based on Al_2_O_3_ with the inclusion of spherical fractions, shaped by uniaxial pressing, as an innovative solution that meets contemporary technological requirements.

#### 2.1.2. Materials and Raw Materials

In the production of refractory ceramic composites, the careful selection of raw materials plays a crucial role, as the quality and performance of the final product largely depend on the physicochemical properties of the components used. In the context of developing a novel ceramic material, particular emphasis was placed on selecting high-purity alumina fractions with tailored characteristics to ensure both refractory performance and mechanical integrity. The material design strategy was based on the assumption that the overall functionality of the composite could be significantly enhanced by engineering the internal porosity distribution. Specifically, the objective was to minimize the open porosity of the fine-grained alumina matrix to ensure high cohesion, while deliberately introducing closed porosity through spherical alumina granules. This targeted differentiation of pore type and spatial distribution was intended to reduce the material’s thermal mass, improve energy efficiency under cyclic thermal conditions, and maintain structural integrity at elevated temperatures.

Fine-grained Al_2_O_3_ powders, CT 300 LS SG (Almatis GmbH, Ludwigshafen, Germany) and Nabalox NO 325 (Nabaltec AG, Schwandorf, Germany), with particle sizes below 2 μm were selected as the main matrix material for the refractory composite. The primary criteria for their selection were high purity (>99.5%) and granulometric properties that would enable the preparation of ceramic mixtures with optimal consistency and facilitate the sintering process [25]. The designations and basic properties of the fine-grained powders used are summarized in Table 1.

An additional material that was introduced into the matrix of the refractory composite was Al_2_O_3_ powder Alodur WRG (IMERYS S.A., Paris, France) with particle size ranging from 1 to 3 mm. The main criteria for its use were the reinforcement of the ceramic matrix (through a crack propagation blocking mechanism [26] and the reduction of shrinkage during the sintering process [27]). The designation and basic properties of the coarse-grained powder are summarized in Table 2.

Al_2_O_3_ spheres Alodur KKW SP (IMERYS S.A., Paris, France) with particle sizes ranging from 0.5 to 1 mm were selected as an innovative raw material to modify the properties of the refractory composite. According to the initial assumptions, the role of the spherical Al_2_O_3_ particles will be to reduce the composite’s density and improve its thermal properties (reduce heat capacity). The number of damaged spheres per volume of raw material and its chemical purity were used as selection criteria. Basic data regarding the properties of the Al_2_O_3_ spheres are summarized in Table 3.

In order to achieve the optimal material formulation and molding capabilities of the granulate, polyethylene wax Lubarit 680 (Munzing Chemie GmbH, Abstatt, Germany) was selected as the raw material fulfilling the function of a lubricating and binding agent. The removal of water from the material formulation eliminated the drying stage and associated possibility of cracking. The use of a binder without the addition of a dispersing agent enabled the preservation of the mold shapes and prevented the material from sticking to the machine components. Basic data regarding the properties of the applied binder are summarized in Table 4.

#### 2.1.3. The Process of Manufacturing the Composite

The technologies for the production of bulk refractories consist of the following basic stages [3]:

- preparation of the slip/mass/granulate
- shaping
- sintering

**Figure 1:**
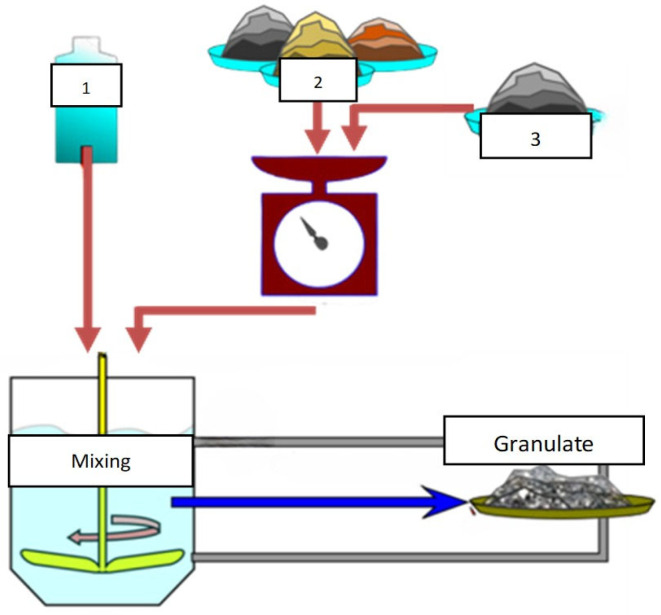
Simplified diagram of ceramic granulate production. 1—binder, 2—fine-grained alumina and coarse-grained alumina, 3—alumina spheres. Red arrows indicate the sequence of processing steps. The blue arrow marks the final formation of the granulate.

In the proposed technological solution for the new refractory ceramic composite (Figure 1), the following assumptions were made:

- elimination of wet/semi-liquid masses → use of granulate to reduce shrinkage
- simplification of shaping through the use of one-step uniaxial pressing

### 2.2. Preparation and Selection of Samples for Investigations

The preparation of refractory composite for the investigations involved production of rectangular samples using the materials specified in Section 2.1.2. These specimens were produced at different uniaxial compression pressures in accordance with the following specifications:

- content of alumina spheres [SG]: 40.80%
- content of fine-grained alumina [MG1]: 20.40%
- content of fine-grained alumina [MG2]: 20.40%
- content of coarse-grained alumina [DG]: 10.20%
- content of binder (Lubarit 680): 8.20%
- pressing pressures: 10, 20, 25, 35, 50 MPa
- sintering temperature: 1700 °C
- sintering time: 39.1 h

The granulate was formed inside rectangular molds with dimensions of 25 × 25 × 150 mm, according to the PN-EN 725-10:2008 standard [28]. The green parts were subjected to visual inspection to detect macroscopic surface and edge irregularities or structural defects—faulted samples were eliminated from further stages of the manufacturing process. Since the only variable in the process was the pressing pressure, a selective sample selection method was applied to reduce the number of tests while ensuring the representativeness of the results.

A selective sample selection method was used in the organization of the research process. This method, also known as purposive sampling [29], involves the deliberate selection of samples that best represent specific characteristics of the material being studied. It allows the exclusion of damaged or heterogeneous specimens that could distort the results of physical and mechanical property analyses. This selection method ensures that all samples tested represent the key characteristics of the new ceramic material based on defined criteria, which consider the research goal—optimization of the refractory composite production technology and its industrial application.

Steps of selection:

1. Defining the research objective: The main goal is to develop a production technology for a new ceramic composite that meets the technical requirements for refractory auxiliary plates.
2. Defining selection criteria: Key criteria include chemical composition, microstructure of the alumina spheres in the composite matrix, required refractory resistance and mechanical strength.
3. Selecting representative samples: Based on the criteria, samples exhibiting the best functional properties, such as porosity, mechanical stability, and structural integrity, were chosen.
4. Characterization of selected samples: This involves comprehensive studies, such as porosity analysis, optical and electron microscopy, and measurement of mechanical and thermal properties. The aim is to obtain a complete picture of the material properties of selected samples.
5. Interpretation and analysis of results: The data obtained from the tests are analyzed in the context of the previously established goals and selection criteria, which allows for the formulation of conclusions regarding further technological improvements.

The purposive sampling method allows for a focus on the most critical technological aspects, enabling effective optimization of the production process and enhancement of the final product’s quality. This approach ensures that the new ceramic material not only improves in quality but also becomes more cost-effective, which is essential for its commercial success and practical application.

#### 2.2.1. Structural Studies

Initially, the samples were analyzed using optical microscopy to assess the overall structure of the material, identify the quantity and distribution of alumina spheres, and detect cracks and other structural defects. The evaluation of the structure of the produced samples was carried out based on microscopic observations (light microscopy and scanning electron microscopy) and X-ray tomography.

Optical microscopy observations were performed using a Keyence VHX-7000 digital microscope (KEYENCE Corporation, Osaka, Japan) equipped with VH-Z100 lenses operating at 100× magnification. Observations were made in co-axial and ring light modes.

Scanning electron microscopy (SEM) observations were performed using a JEOL JSM-6610LV SEM microscope (JEOL Ltd., Tokyo, Japan) with an X-MAXN 80 EDS (Oxford Instruments, High Wycombe, UK) attachment. Observations were made in secondary electron (SE) mode under high vacuum conditions (~1 Pa) with an accelerating voltage of 10–15 kV. The surfaces of the samples were previously coated with a thin gold layer using vacuum sputtering to improve conductivity.

The internal structure of the material was investigated using X-ray computed tomography (CT) using GE Phoenix v|tome|x S240 device (General Electric, Boston, MA, USA). CT device allows radiological analysis of objects up to 260 × 420 mm in size. The samples were extracted using a diamond-tipped saw and scanned in 360° mode with increments of less than 1°, and scanning resolution of less than 0.2 μm.

#### 2.2.2. Porosity Studies

The distribution and size of pores was analyzed by mercury intrusion porosimetry using TriStar 3000 porosity analyzer (Micromeritics, Norcross, GA, USA). The measurement involved injecting liquid mercury into the pore spaces of ceramic samples pressed at 25 MPa. The analysis was conducted with a wetting angle of 130° and a mercury surface tension of 485 mN/m. Based on the intrusion pressure values and the volume of mercury injected, the total pore volume, pore size distribution, and characteristic pore diameter were determined.

#### 2.2.3. Mechanical Studies

The flexural strength tests were conducted according to PN-EN 843-1:2007 [30] using a Zwick Roell Z020 universal testing machine (Zwick Roell Group, Ulm, Germany). The samples, measuring 25 × 25 × 150 mm, were subjected to three-point bending until fracture. The maximum destructive force applied to the sample was recorded, and the flexural strength (Rg) was determined. The tests used 15 samples for each of the five pressing pressure levels: 10, 20, 25, 35, and 50 MPa.

Compression strength tests were performed according to PN-EN 993-5:2001 [31] using the ToniNORM testing machine (ToniTechnik Baustoffprüfsysteme GmbH, Berlin, Germany). The machine was equipped with two DD1 electronic extensometers from HBM, allowing precise measurement of the sample’s deformation during loading. The tests were performed on cylindrical samples with dimensions of 64 × 64 mm, specially prepared for this test. Ten samples were used for each of the pressing pressures: 10, 20, and 25 MPa. Samples pressed at higher pressures (35 and 50 MPa) were excluded from testing due to post-processing cracks, disqualifying them from further analysis.

#### 2.2.4. Refractoriness Studies

Refractoriness under load was evaluated according to PN-EN ISO 1893:2009 [32] using a Netzsch GmbH R.U.L./C.I.C 421 thermomechanical analysis device (Netzsch GmbH, Selb, Germany). The samples in cylindrical form (φ = h = 50 mm) were prepared by pressing at 25 MPa and then sintered at high temperature, following the procedure described in Section 2.2. The refractoriness under load test determined the softening temperature of the material, T_0.6_, at which the sample undergoes a 0.6% deformation of its initial size, and T_4_, the temperature at which the sample undergoes a 4% deformation. The test was conducted in an air atmosphere with a heating rate of 5 °C/min, as per PN-EN ISO 1893:2009 requirements.

## 3. Results and Discussion

### 3.1. Morphology

The microstructure observations of samples were conducted using SEM and optical microscopy to assess the shape and distribution of alumina spheres and the degree of porosity in the matrix. Special attention was paid to the presence of intact spheres, the level of porosity in the matrix, and any particle damage resulting from the forming process under different pressure conditions.

**Figure 2:**
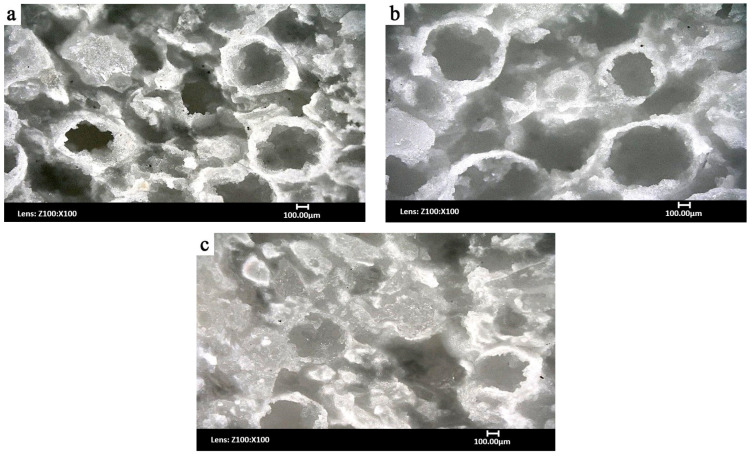
Image of the ceramic composite formed under a pressure of 10 MPa (**a**), 20 MPa (**b**), and 25 MPa (**c**); sample view obtained using an optical microscope. Magnification 100×.

In Figure 2a, the morphology of the composite formed at 10 MPa is shown. Numerous well-preserved spheres with a regular, circular shape are visible, randomly distributed throughout the material. The porosity in the matrix is clearly present and arranged irregularly. Damaged spheres are also noticeable, mainly as a result of cutting during sample preparation.

Figure 2b shows microstructure of a sample formed at 20 MPa. Compared to the sample formed at 10 MPa (Figure 2a), the porosity is lower, and the matrix structure is denser. The alumina spheres are more closely packed, with no visible signs of deformation. Additionally, large, intact polyhedral corundum crystals are visible, which can be crucial in terms of inhibiting crack propagation.

For the sample formed at 25 MPa (Figure 2c), the composite structure becomes even more compact, with minimal porosity in the matrix. The alumina spheres retain their shape, but the number of intact particles decreases, which may indicate the beginning of their destruction under excessive forming pressure. These observations suggest that the optimal forming pressure range for the analyzed ceramic composite lies between 10 MPa and 25 MPa, allowing for sufficient material densification without compromising the integrity of the dispersed phase particles.

SEM observations provided rapid and direct identification of potential technological errors post-sintering. Unlike other methods, samples did not require time-consuming preparation, enabling regular control of each stage of the composite forming process.

**Figure 3:**
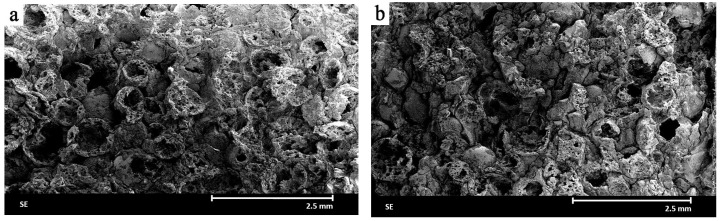
Images of the microstructure of the ceramic composite formed under a pressure of: (**a**) 10 MPa and (**b**) 20 MPa, obtained using a scanning electron microscope (SEM).

In Figure 3, SEM microstructures of the ceramic materials formed at 10 MPa and 20 MPa are shown. In both cases, well-preserved circular alumina spheres are visible, indicating that low pressure forming does not lead to destructive effects on the material. However, significant porosity is observed both in the matrix and along the edges of the cut spheres. Discontinuities in the bonding between the spheres and the matrix, manifested as delaminations, are also visible, which may negatively impact the material’s mechanical properties.

**Figure 4:**
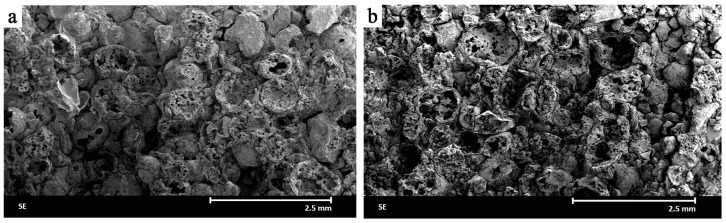
Images of the microstructure of the ceramic material formed under a pressure of 25 MPa (**a**) and 35 MPa (**b**), obtained using a scanning electron microscope (SEM).

Increasing the forming pressure to 25 MPa (Figure 4a) results in significant changes in the structure of the composite. The material becomes denser, and the number of open pores clearly decreases. The alumina spheres retain their original shape and are better surrounded by the ceramic matrix, improving the integrity of the material. Similarly to the optical microscopy analysis (see Figure 2c), large, sharply pointed corundum crystals are visible, serving as barriers to the propagation of microcracks.

Further increase in forming pressure to 35 MPa (Figure 4b) results in noticeable morphological changes. Some of the spheres have deformed—their shape becomes flattened and irregular, and the number of undamaged structures decreases. These observations led to the exclusion of higher forming pressures (above 25 MPa) from further analyses. Pressing at 35 MPa and above resulted in mechanical damage to the alumina spheres, which disrupted the structural integrity of the composite and significantly reduced the amount of desired closed porosity. As the presence of intact spherical pores was a key design feature for reducing thermal mass while maintaining strength, the loss of this structural element was considered detrimental to the material’s functional properties. These changes indicate exceeding the limiting forming pressure above which mechanical damage to the alumina spheres occurs. Despite the compacted structure, excessive pressure causes degradation of this fraction, which may affect the long-term strength of the material.

In summary, the best morphological properties were obtained for samples formed under a pressure of 25 MPa. On one hand, this pressure allowed for proper densification of the matrix, and on the other hand, it did not yet lead to the destruction of the alumina spheres. The SEM observations turned out to be fully consistent with the optical microscopy analyses.

The X-ray computed tomography was applied only to the ceramic composite sample formed under 25 MPa, which—according to previous microscopic observations—exhibited the best morphological integrity. Non-destructive CT method allowed for the analysis of the internal structure of the material without the need to compromise its continuity.

**Figure 5:**
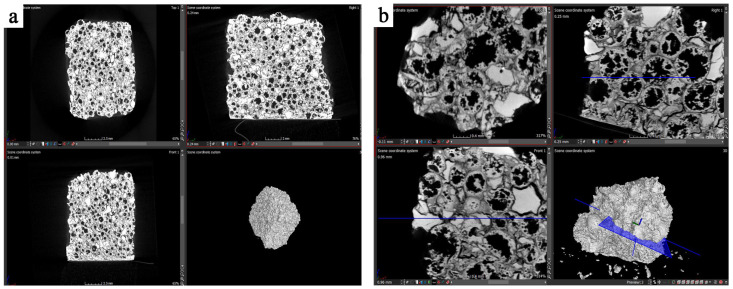
Image from the analysis of the ceramic composite using X-ray tomography with a resolution of 18.5 µm (**a**) and 6.5 µm (**b**).

Two CT scans were conducted: the first with a resolution of 18.5 µm (Figure 5a) and the second—more detailed—with a resolution of 6.5 µm (Figure 5b).

The images obtained from the first scan (Figure 5a) revealed the presence of undamaged alumina spheres distributed throughout the entire volume of the sample. Alumina spheres retained their spherical shape, indicating no deformation during the technological process. The open porosity of the matrix is also visible, arranged irregularly, but present throughout the entire cross-section of the sample. The CT images also showed large particles of aluminium oxide, which, according to the structural design of the material, could act as crack barriers.

The more detailed scan (Figure 5b) allowed for a more detailed assessment of the morphology. It was confirmed that the spheres are tightly surrounded by the ceramic matrix, strengthening the composite structure. An important observation is the variation in the thickness of the alumina sphere walls, which can influence local stresses and consequently the strength of the material. The interiors of the spheres exhibit high roughness and heterogeneity, which may be relevant in the context of cracking and thermal conductivity.

In any of the scans, no cracks indicating destructive effects from the forming process were observed. The alumina spheres and large corundum particles were randomly distributed and present throughout the entire cross-section of the sample. In summary, X-ray CT confirmed that the process of producing the ceramic composite based on spherical alumina does not cause damage to its internal structure. The material is characterized by the presence of both open and closed porosity, which may influence its functional properties.

In order to evaluate the porosity and distribution of pores, porosimetry tests of sintered 25 MPa sample was conducted. The results of the test are presented in Table 5.

The obtained results indicate that the sample exhibits an open porosity of 47%. Within the volume of the material, the largest contribution to porosity came from pores with a diameter of 1–10 µm and 10–20 µm, which accounted for 39% and 35.9%, respectively. Pores larger than 20 µm constituted about 22% of the material’s volume, while the smallest pores with a diameter of 0.25–1 µm accounted for 3% of the volume. The median pore diameter was 2.05 µm, and no pores smaller than 0.25 µm were found in the material.

**Figure 6:**
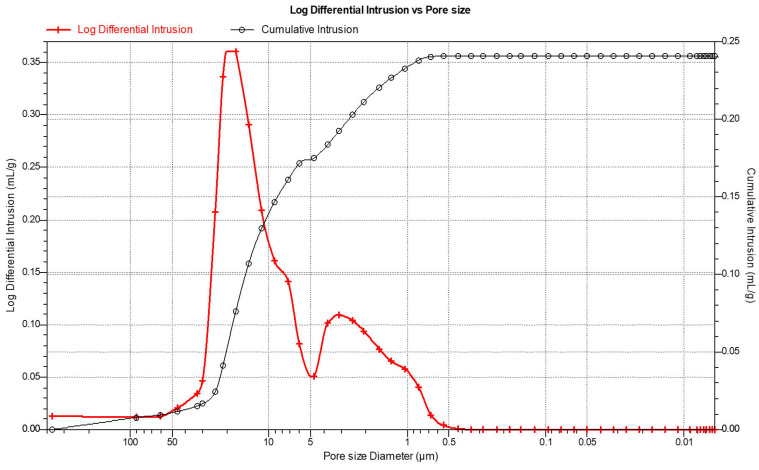
Graphical representation of the results of the study on the distribution and porosity distribution of the ceramic composite.

In addition, an analysis of the pore distribution based on the measurement of mercury intrusion into the open porosities was also carried out. The analysis software (Micromeritics, Norcross, GA, USA) allowed for the presentation of the open pores volume as a function of the applied pressure. The pore sizes correspond to specific pressure values, which allowed for the determination of their distribution. Results are shown in Figure 6. Due to the large variation in pore sizes (from the smallest to the largest), the results were presented in a logarithmic scale in reverse order, starting from the largest values down to the smallest.

The mercury porosimetry method allowed not only the determination of the material’s porosity but also the quantitative contribution of pores classified according to their diameter. Figure 6 presents the pore size distribution curve of the ceramic composite material, determined using mercury intrusion porosimetry. The graph shows the logarithmic differential intrusion as a function of pore diameter. The distribution exhibits a clear multimodal character, indicating the presence of pores with various diameters across the structure.

The primary peak, located within the range of approximately 1–10 µm, indicates the dominant pore size range in the tested material. This region corresponds to the highest volume of mercury intrusion, suggesting a significant contribution of medium-sized pores to the overall porosity. A secondary, less pronounced peak is observed around 20–30 µm, which may be associated with intergranular voids or structural defects resulting from incomplete compaction or burn-out of the organic binder.

The curve sharply decreases beyond 50 µm, indicating that large pores are relatively rare in the material. Below 1 µm, the intrusion volume is negligible, suggesting the absence of significant amounts of nanopores.

The pore size distribution confirms that the pressing and sintering process applied under 25 MPa results in a material structure dominated by medium-sized open pores, which is consistent with previous microstructural observations and porosity measurements.

### 3.2. Mechanical Properties

#### 3.2.1. Bending Strength

**Figure 7:**
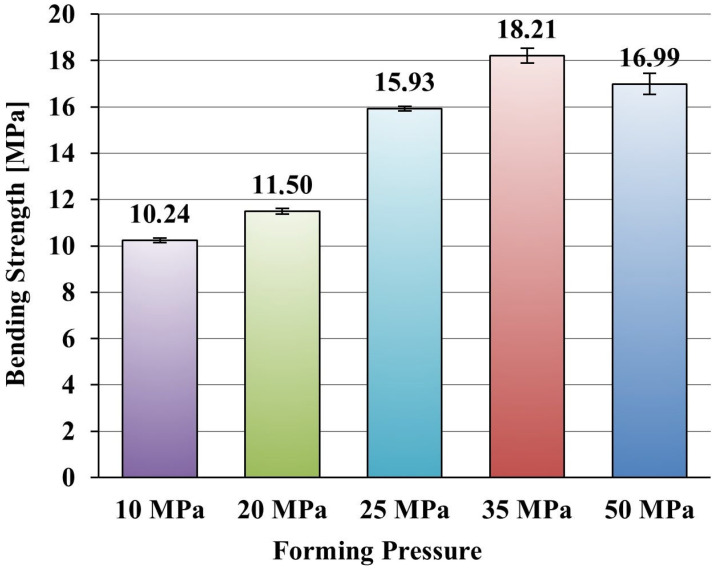
Summary of bending strength test results of the ceramic composite for different molding pressures.

The results of the bending strength tests of the ceramic samples are presented graphically in Figure 7.

Samples produced at 10 and 20 MPa forming pressures exhibited significantly lower bending strength compared to samples formed at higher pressures. A possible reason for this phenomenon could be the excessive presence of open porosity, which was not sufficiently compacted during the forming. Such result indicate relationship between porosity and bending strength: as the porosity of the material increases, the bending strength of the ceramic samples decreases. The best bending strength was observed in samples formed at pressures above 35 MPa. However, technological issues related to the production of the ceramic composite, such as the cracking of spherical alumina particles at higher pressures, negatively impacted the bending strength results.

Analysis of the results shows that samples pressed at pressures of 25 and 50 MPa exhibited similar bending strength, approximately 16 MPa. The highest bending strength was obtained for samples pressed at 35 MPa. These results suggest that microcracks in the material structure can be effectively dispersed on the alumina spheres or large corundum crystals introduced into the structure to block crack propagation. In the case of samples pressed at the highest pressure of 50 MPa, the material’s bending strength began to decrease. This could indicate that excessive forming pressure reduced the number of crack propagation barriers in the material volume, which could have resulted in the absence of alumina spheres in the area of the specimen where the force was applied.

#### 3.2.2. Compressive Strength

The compressive strength of the ceramics, as an indicator of the material’s resistance to mechanical impact, is a key property in the context of applications in the high-temperature industry, including the production of refractory plates. The average compressive strength results of samples compacted at 10, 20 and 25 MPa are presented in Table 6.

The lowest compressive strength was observed for the material formed at the lowest pressing pressure 10 MPa, which was related to insufficient compaction of the ceramic material. In contrast, the highest compressive strength (9.37 MPa) was shown by the material pressed at 25 MPa, which is exceptionally high for a material with high porosity. During the testing, a large variability in individual compressive strength results was noted, especially in samples pressed at the extreme pressures of 10 and 25 MPa.

The tested samples, which were dedicated to compression testing, were not used for other tests, thus eliminating the possibility of defects caused by damage from other samples or analyses. The obtained compressive strength results for the series of samples formed at 10 and 20 MPa, in correlation with the earlier results, led to a conscious elimination of material variants in the further stages of the research, in favor of samples formed at 25 MPa. Adopting this research approach was consistent with the purposive sample selection method.

#### 3.2.3. Refractoriness

The results of the refractoriness of the ceramic composite under the standard load of 0.2 MPa for samples pressed at 25 MPa are presented in Table 7.

For all tested samples, the temperature T_0_._6_, which indicates the start of material softening, was 1500 °C. For the next critical point, the T_4_ temperature, the samples also showed a uniform value of 1670 °C. At this temperature, the height of the tested samples decreased by 4% compared to the initial dimensions. The value of the T_4_ temperature allows the approximate maximum “under load” temperature for refractory plates made of the new ceramic material to be determined at 1670 °C. These results provide crucial information for establishing the temperature range in which the refractory material can be used in industrial conditions, especially in high-temperature processes, where it must maintain its mechanical and structural properties.

## 4. Conclusions

Based on the results of the study, the following conclusions can be drawn:

1. A forming pressure of 25 MPa provides the best balance between porosity and mechanical strength, achieving superior flexural (16 MPa) and compressive (9.37 MPa) strengths. This pressure minimizes defects while maintaining structural integrity, making it appropriate for producing high-quality ceramic composites.
2. Porosity significantly influences the material’s performance. Higher porosity at lower pressures (10–20 MPa) compromises mechanical strength, while lower porosity at higher pressures (35–50 MPa) enhances strength but risks cracking due to processing defects.
3. The use of spherical alumina (40.80% by weight) and a dual-particle-size alumina matrix (0.5–2.0 μm) enhances the material’s microstructure and mechanical properties. Eliminating the water dispersant simplifies the manufacturing process by removing the drying stage, reducing shrinkage and improving production efficiency.
4. The designed microstructure allowed the preservation of closed porosity introduced by the spherical alumina granules. This feature contributed to lowering the thermal capacity of the material while maintaining mechanical strength, which is particularly advantageous in applications with cyclic thermal loading.
5. The ceramic composite’s ability to withstand temperatures up to 1670 °C under mechanical loading highlights its excellent thermal stability, making it a strong candidate for applications in metallurgy, glass production, or other high-temperature processes.
6. The material’s combination of mechanical strength, thermal stability, and optimized manufacturing process makes it particularly suitable for refractory auxiliary plates and other components in extreme-temperature environments.

### Future Considerations

○ While 25 MPa is optimal, further research could explore mitigating cracking at higher pressures (e.g., 50 MPa) to potentially enhance mechanical properties without sacrificing structural integrity.
○ Additional studies could investigate the long-term durability of the material under cyclic thermal and mechanical loading to confirm its reliability in industrial settings.

In summary, a high-performance ceramic composite using spherical alumina and an optimized uniaxial pressing process at 25 MPa was successfully developed. The material’s excellent mechanical properties and thermal stability position it as a promising solution for high-temperature industrial applications, particularly in refractory systems.

## Figures and Tables

**Table 1:** Chemical composition, physical properties, and designations of CT 300 LS SG and Nabalox NO 325 alumina powders.

	CT 300 LS SG	Nabalox NO 325
Chemical composition:		
Al_2_O_3_ [%]	99.80	99.70
Na_2_O [%]	0.03	0.30
Fe_2_O_3_ [%]	0.015	0.03
SiO_2_ [%]	0.015	0.03
Specific surface area [m^2^/g]	7.80	1.50
Average grain size [μm]	0.50–2.00	2.00
Specific density [g/cm^3^]	3.95	3.90
Designation	MG1	MG2

**Table 2:** Chemical composition, physical properties, and designation of Alodur WRG alumina powder.

	Alodur WRG
Chemical composition:	
Al_2_O_3_ [%]	99.78
Na_2_O [%]	0.24
Fe_2_O_3_ [%]	0.04
Average grain size [μm]	1.00–3.00
Specific density [g/cm^3^]	3.70–4.50
Melting point [°C]	2050
Designation	DG

**Table 3:** Chemical composition, physical properties, and designation of Alodur KKW SP.

	Alodur KKW SP
Chemical composition:	
Al_2_O_3_ [%]	98.80
Na_2_O [%]	0.10
Fe_2_O_3_ [%]	0.03
SiO_2_ [%]	0.10
MgO [%]	0.01
CaO [%]	0.03
Average grain size [μm]	0.50–1.00
Specific density [g/cm^3^]	0.80–1.00
Melting point [°C]	2050
Designation	SG

**Table 4:** Chemical composition and physical properties of Lubarit 680 binder.

	Lubarit 680
Chemical composition	polyethylene wax
Melting range [°C]	98
pH level	10
Viscosity [mPas]	15–35

**Table 5:** Results of a study on pore distribution in ceramic composite.

Tested Parameter	Unit	Result
Mercury intrusion volume	mL/g	0.241
Total surface area	m^2^/g	0.196
Median volume pore diameter	µm	12.43
Median surface pore diameter	µm	2.05
Open porosity	%	47.13
Permeability	Mdarcy	120.1
Pore tortuosity	-	6.6
Distribution of pore sizes		
>90 µm	%	3.25
60–90 µm	%	0.75
30–60 µm	%	3.05
20–30 µm	%	14.97
10–20 µm	%	35.90
1–10 µm	%	39.06
0.5–1 µm	%	2.96
0.25–0.5 µm	%	0.06
0.1–0.25 µm	%	0.00
Sum	%	100.00

**Table 6:** Compressive strengths of the ceramic composites formed at compacting pressures of 10, 20 and 25 MPa.

Forming Pressure	Compressive Strength [MPa]
10 MPa	8.35 ± 0.21
20 MPa	8.61 ± 0.24
25 MPa	9.37 ± 0.19

**Table 7:** Refractoriness of ceramic composites formed at 25 MPa compacting pressure.

Sample Number	T_0.6_ [°C]	T_4_ [°C]
1	1500	1670
2	1500	1670
3	1500	1670
Average	1500	1670

## Data Availability

The original contributions presented in this study are included in the article. Further inquiries can be directed to the corresponding author.

## Abbreviations

The following abbreviations are used in this manuscript:

Al_2_O_3_	Aluminium oxide
CT	Computed Tomography
DG	Coarse-Grained Alumina (Alodur WRG)
EDS	Energy Dispersive X-ray Spectroscopy
ISO	International Organization for Standardization
MG1	Fine-Grained Alumina (CT 300 LS SG)
MG2	Fine-Grained Alumina (Nabalox NO 325)
MPa	Megapascal
PN-EN	Polish/European Norm
RUL	Refractoriness Under Load
SE	Secondary Electrons
SEM	Scanning Electron Microscopy
SG	Spherical Granules (Alodur KKW SP)
X-ray CT	X-ray Computed Tomography
